# Enzymatic Crosslinking of Amino Acids Improves the Repair Effect of Keratin on Hair Fibre

**DOI:** 10.3390/polym15092210

**Published:** 2023-05-06

**Authors:** Yang Liu, Jingjing Liu, Jing Xiao

**Affiliations:** State Key Laboratory of Biobased Material and Green Papermaking, Qilu University of Technology, Shandong Academy of Sciences, Jinan 250353, China

**Keywords:** glutamine transaminase, wool hydrolysed keratin, serine, hair, crosslinking, repair

## Abstract

Although keratin can effectively repair hair fibres and enhance their moisture content and flexibility, it has a relatively low affinity for hair. In this study, the effects of transglutaminase (TGase)—commonly used to catalyse crosslinking of proteins or amino acids—in crosslinking serine and hydrolysed wool keratin to repair damaged hair and protect healthy hair were studied. Treatment with a repair solution containing hydrolysed wool keratin, serine, and TGase improved the physical and chemical properties of damaged hair samples. The alkali solubility of damaged hair samples decreased by 50.53%, fracture stress increased from 1.031 to 1.806 N, and fracture strain increased from 9.51 to 19.88 mm. Fourier transform infrared spectroscopy and X-ray analysis showed that amide bonds increased in damaged hair samples treated with the repair solution and hair crystallinity increased. Differential scanning calorimetry showed that the repair solution improved the thermal stability of damaged hair. After five cycles of washing, the effects of the repair solution were still apparent in damaged hair samples. The enzymatic solution had stronger repair effects than general hair care products and reduced water loss rates in damaged hair samples; repaired hair samples were also softer and brighter. The repair solution was effective in protecting healthy hair samples against chemical damage. The materials used to prepare the repair solution are all bio-based, and treatment with this product is safer and longer lasting.

## 1. Introduction

Hair fibres comprise the cuticle, cortex, and medulla [1], and the main component of hair fibres is keratin (65–95% by weight) [2]. The stratum corneum is covered with scales, which can reduce mechanical damage. The cortical layer is an important part of the hair, and the thickness of the hair is mainly determined by the cortical layer. The cortex layer contributes to mechanical stability, and there is an adhesion layer between the stratum corneum and the cortex, called the cell membrane complex (CeMeCo) [3]. The outermost stratum corneum is vulnerable to various physical and chemical insults, a structurally intact CeMeCo prevents the stratum corneum from falling off and helps avoid damage to the internal cortical layer.

There are many causes of hair fibre damage, including mechanical damage from combing and stretching. Perming and other hair treatments can cause chemical damage; disulphide bonds in hair are easily reduced by thiols in the chemical reagent [4], accompanied by dissolution of hair protein components and structural damage, leading to loss of hair gloss, dryness, and easy splitting. Under these circumstances, the elasticity, toughness, and strength of hair are reduced. Maintenance and repair of hair is one of the important needs of people in pursuit of a high-quality and healthy life.

Today’s environmental concerns and environmentally friendly attitudes have led people to pursue more eco-friendly hair care products [5]. Hair displays dual properties of protein and amino acids, and both proteins and amino acids can be used for hair care [6]. Many current hair care products include various animal or plant protein hydrolysates or amino acid products [3]. Keratin and the corresponding hydrolysate can effectively repair hair fibre and enhance the moisture content and flexibility of hair [7]. Barba et al. [8] studied the use of wool keratin peptides and proteins in hair bleaching to improve the water absorption of hair fibres and reduce their permeability. Villa Ana et al. [9] studied the effect of feather keratin hydrolysate on hair fibres and showed that keratin peptides were deposited at the junction of the stratum corneum, with benefits for hair care. However, keratin has a relatively low affinity for hair. Amino acids such as serine have a level of affinity for hair and are nutritionally active substances as well as being moisturising ingredients with the ability to help repair damaged hair. Some hair conditioners on the market contain added amino acids, but the concentrations are low, and the amino acids rinse off easily.

Maintenance and repair of hair should not merely target a short-term effect but should aim to generate a benefit that is stable over the long term. Transglutaminase (TGase) is commonly used to catalyse crosslinking of proteins or amino acids [10]. It has been applied in the processing and production of some protein fibres to improve product quality. Soun Bhawna et al. [11] found that scales of wool fibres treated with transglutaminase were smoother and flatter; compared with those of untreated scoured wool fibres, their softness, lustre, and tensile strength were improved. Jeanette M [12] applied TGase and keratin hydrolysate to wool fabrics to modify keratin-based biomaterials. Both human hair and wool are mainly composed of α-keratin, and the amino acid content of each fibre type is similar [11]; therefore, results for TGase in wool repair may also be expected to apply to human hair [13]. Lin et al. [14], through the mTG enzyme, found that the amino acids with homology are bonded with the amino acid residues on the molecular chain of the wool fabric and, simultaneously, the macromolecular chains of the broken wool fibres are re-bonded and crosslinked, so that the performance of the fragile wool fabric is improved. In 2021, Li Ziyuan et al. [15] used low-concentration keratin with the TG enzyme to directionally catalyse more covalent crosslinking of keratin to repair damaged hair samples. In this study, we aimed to test the hair care effects of crosslinking amino acids and proteins using TGase.

## 2. Materials and Methods

### 2.1. Materials

Hair samples were gathered from healthy Chinese adult women with no history of perms.

Wool hydrolysed keratin (0.8–1 kDa, Fourbon Biology. Shanxi, Xi’an, China), TG enzyme (enzyme activity 6000 U/g, Shandong Longke Enzyme Preparation Co., Ltd. Linyi, China), sodium hydroxide (analytical grade, Chemical Reagent of National Pharmaceutical Group Co., Ltd. Shanghai, China), perming agent (250 g/bottle, containing mercaptoethanol and ammonia. Tianjin Xinli household necessities factory, Tianjin, China), serine (purity > 99%, Beijing Kulaibo Technology Co., Ltd. Beijing, China)

### 2.2. Repair Solutions

Keratin–enzyme repair solution(K-eRS) contained 5% (*w*/*v*) hydrolysed wool keratin and 150 U mL^−1^ TGase in water. Keratin-serine repair solution(K-sRS) contained 5% (*w*/*v*) hydrolysed wool keratin and 7.5% (*w*/*v*) serine. Keratin-serine–enzyme repair solution(K-s-eRS) contained 5% (*w*/*v*) hydrolysed wool keratin, 7.5% (*w*/*v*) serine, and 150 U mL^−1^ TGase.

### 2.3. Preparation of Permed Hair Samples

Hair samples approximately 30 cm in length, with a weight of approximately 0.1 g, were soaked in 20 mL perm solution (containing mercaptoethanol, ammonia water) for 30 s, shaped in an oven at 65 °C for 50 min, washed with deionised water, and dried to constant weight.

### 2.4. Hair Repair

Permed hair samples were immersed in the different repair solutions in a water bath at 40 °C for 30 min, washed with deionised water, and then, dried to constant weight.

### 2.5. Characterisation of Amide Bonds (FTIR)

Dry hair samples were cut into pieces and subjected to attenuated total reflectance–Fourier transform infrared spectroscopy (IR Prestige-21, Shimadzu, Japan). Spectra were obtained using 16 scans at a resolution of 4 cm^−1^.

### 2.6. Determination of Hair Crystallinity (XRD)

Treated hair samples were cut into pieces, placed on the sample stage, and subjected to X-ray diffractometer (Japan Science Smartlab SE, Tokyo, Japan) continuous scanning over a scanning range of 5–90°. The common diffraction peak (I_1_) for α-helices and β-sheets occurs close to 9°, whereas α-helix diffraction peaks occur at 15–30°, and β-sheet diffraction peaks occur at 16–30° (I_2_). A published method [16] was used to determine the relative crystallinity of treated hair samples.


$$\mathrm{CI}(\%)=\frac{I_{1}-I_{2}}{I_{1}}$$

### 2.7. Determination of Alkali Solubility

Treated hair samples were added to 100 mL 0.2 M NaOH and held at 65 °C for 1 h. The reaction was filtered, and the filtrate was washed with distilled water or dilute acetic acid and then dried to constant weight in an oven at 65 °C [17]. Three parallel experiments were carried out for each repair solution. Calculation of alkali solubility was based on the weight change in the hair sample before and after alkali treatment:

$$a=\frac{X(1-G)-W}{X(1-G)}\times 100$$

where X is the weight of the hair sample before alkali treatment, G is the moisture content (close to 0%), and W is the mass of the remaining sample after alkali treatment.

### 2.8. Assessment of Thermal Stability (DSC)

Thermal stability of hair samples was assessed using differential scanning calorimetry (DSC) (DSC Q2000, TA Instrument, New Castle, DE, USA). Treated hair sample (approximately 10 mg) was placed in a sealed aluminium crucible and heated from 0 to 300 °C at a heating rate of 10 °C min^−1^ and a nitrogen flow rate of 50 mL min^−1^ to obtain a DSC spectrum.

### 2.9. Determination of Mechanical Properties

An electronic fibre-strength-testing machine was used for hair sample strength testing in accordance with the GB/T 3923.1-1997 standard. After each hair sample was dried, 10 samples were selected at random, and strength testing was conducted on the middle section of the hair sample. The clamping distance was 3 cm, and the tensile rate was 3 mm min^−1^ [14]. After removal of outlier values, the average of the remaining results was taken to represent the fracture stress and fracture strain of the hair sample.

### 2.10. Morphological Characterisation (SEM)

Treated hair samples were coated with gold by sputtering, fixed to a sample stage with conductive adhesive, and subjected to scanning electron microscopy (Hitachi REGULUS8220, Tokyo, Japan) using an observation voltage of 3.00 kV.

### 2.11. Damage Prevention Testing

Healthy hair samples were treated with the three repair solutions and then perm-treated. The alkali solubility of healthy hair samples subjected to repair treatment was compared to that of hair samples subjected to perming without pre-treatment.

### 2.12. Hair Care Durability Test

Damaged hair samples were treated with the three repair solutions and then washed and dried to constant weight, and repeated five times, followed by alkali solubility testing. The durability of each repair solution was determined by comparing the alkali solubility for the treated samples after five cycles with that of the same hair sample after a single cycle of washing and drying.

### 2.13. Moisturising Performance Test

Untreated hair samples and hair samples treated with the three repair solutions were dried to constant weight in an oven. Saturated KCl solution was used to hold the relative humidity in a desiccator at room temperature to 80%. Hair samples were transferred into the desiccator and reweighed after 24 h (m_1_). The relative humidity in the desiccator was then adjusted to 43% using saturated K_2_CO_3_ solution, and the hair samples were placed in it. After 24 h, samples were again weighed (m_2_), and the water loss rate (R) was calculated.


$$R=\frac{m_{1}-m_{2}}{m_{1}}\times 100\%$$

## 3. Results and Discussion

### 3.1. Formation of Amide Bonds

**Figure 1:**
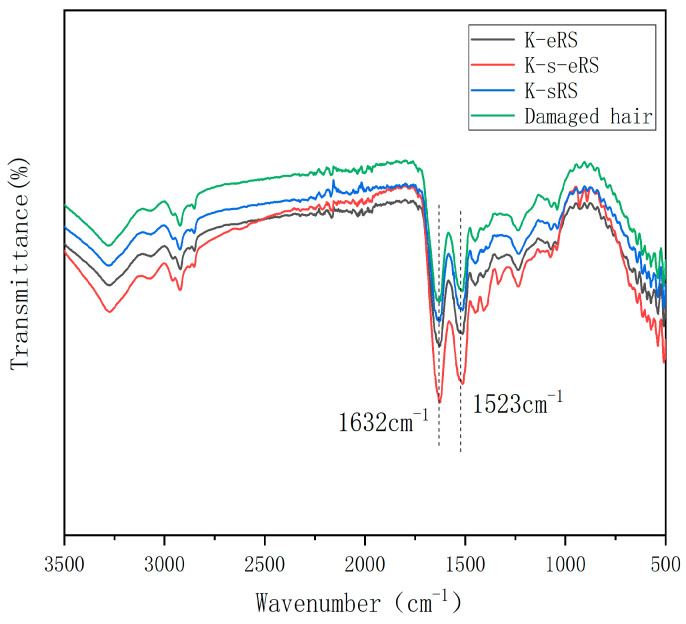
Infrared spectra of damaged and repaired hair.

Keratin-serine plus enzyme repair solution was used to repair the damaged hair samples. Although the secondary structure of hair was not changed, on the one hand, the TG enzyme could promote the interweaving of keratin and serine into a network. On the other hand, it promoted the crosslinking of keratin-serine plus enzyme with the hydrophilic groups exposed in the hair structure, especially in the damaged parts, and increased the amide bond of hair protein, the interaction force between the keys is strengthened, the toughness of the hair is increased, and the occurrence of the phenomena of fragility and fragility after the hair is scalded and damaged is reduced, as shown in Figure 1.

After the addition of serine, the peak area of the amide band at a wavelength of about 1600~1500 cm^−1^ was the largest in the damaged hair sample, which proved that the keratin-serine plus enzyme repair solution was used to repair the damaged hair sample efficiently [18]. Although the band area of the keratin plus enzyme repair hair sample was larger than that of the damaged hair sample, the amplitude was much smaller than that of the keratin-serine plus enzyme repair hair sample, indicating that the effect of adding serine was better. The peak area of the specific amide band of the keratin-serine repair hair sample was only slightly increased compared with that of the damaged hair sample, indicating that the effective component of the hair care was weakly bound to the damaged part and lost in the cleaning, which proved the importance of the catalytic crosslinking of the TG enzyme.

Keratin–enzyme repair solution(K-eRS). Keratin-serine repair solution(K-sRS). Keratin-serine–enzyme repair solution(K-s-eRS).

### 3.2. Hair Crystallinity

**Figure 2:**
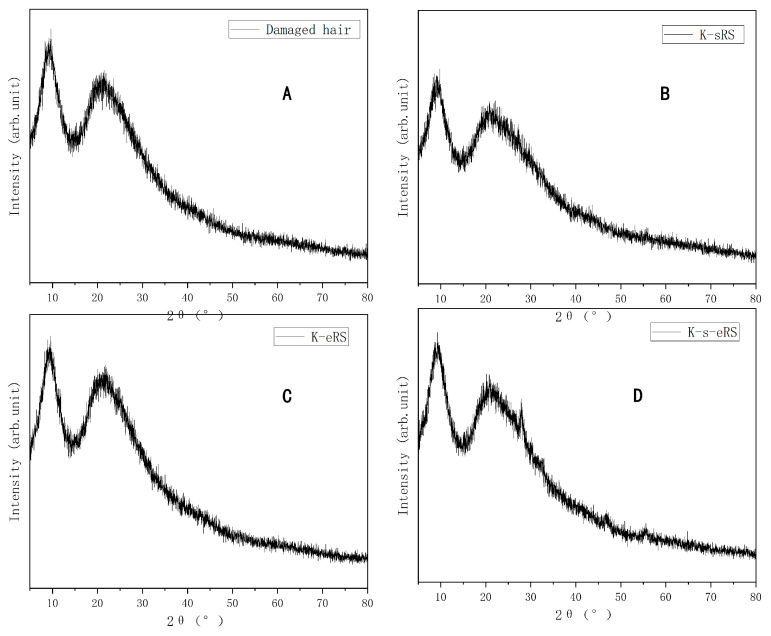
X-ray diffraction pattern of damaged hair and treated samples (**A**–**D**): (**A**) Damaged hair; (**B**) K-sRS; (**C**) K-eRS; (**D**) K-s-eRS.

According to the formula, the crystallinity of each sample was calculated. In the damaged hair sample, the crystallinity of the hair sample treated with keratin-serine plus enzyme repair solution increased by 37.20% compared with the damaged hair sample; compared with the hair sample of the keratin-serine repair solution, the crystallinity increased by 31.49%; compared with the hair sample treated with the keratin plus enzyme repair solution, the crystallinity increased by 24.31%, as shown in Table 1 and Figure 2.

The crystallinity of hairy fibres treated with keratin-serine plus enzyme repair solution increased, and the structure of the hairy protein was more orderly and dense. Combined with the infrared spectrum of hair samples, it can be explained that TG enzyme crosslinking serine and keratin is beneficial to the improvement of the hairy structure. The improvement in hair structure, where the orderliness of the hair sample structure is increased, the hair sample structure becomes denser, the injury resistance is enhanced, and it is more difficult for chemicals to enter, in theory, will reduce the alkali solubility, so that the fibre tensile strength, toughness, and stability are improved, is conducive to reducing the external physical and chemical damage [19].

### 3.3. Alkali Solubility

**Figure 3:**
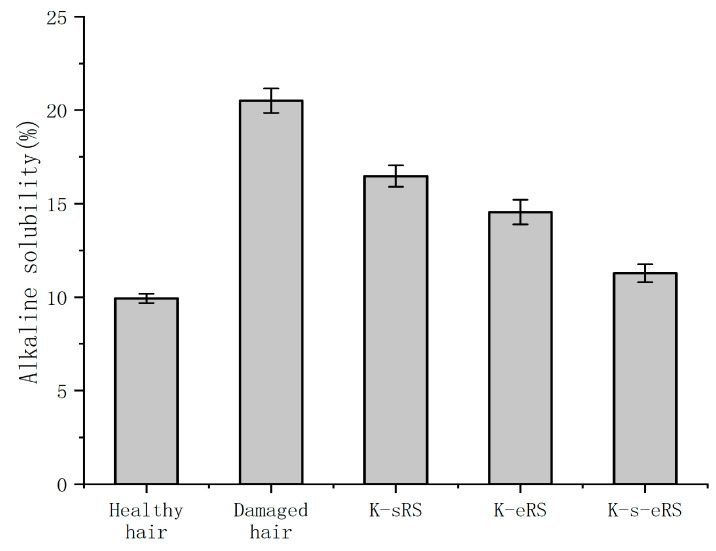
The alkaline solubility of permed hair sample and treated hair sample.

The more serious the hair damage, the higher the alkali solubility. The hair structure after cold blanching was damaged, and the damage could be quantitatively detected by measuring the alkali solubility in alkaline solution, as shown in Figure 3. The internal structure of damaged hair was easier to contact with the alkali solution, so the alkali solubility increased. Compared with the alkali solubility of healthy hair, the alkali loss of cold perming hair reached 52.6%. The keratin-serine mixture can repair the damaged hair sample to a certain extent, which reduces the alkali solubility by 25.40% compared with the damaged hair sample. However, keratin and serine are easy to be lost during the cleaning process, and the anti-alkali protection effect is limited. In the keratin and enzyme repair solution, TGase leads to keratin having a stronger binding ability with the damaged parts through amide bonds, repairing damaged structures, and reducing alkali solubility by 36.86%. Further optimization of keratin-serine plus enzyme repair solution is based on keratin plus enzyme, and serine is added. The addition of serine repairs more damaged parts of macromolecular keratin that are not repaired to form more amide bonds. At the same time, the crystallinity of the hair is improved, and the solvent resistance of the fibre is enhanced. The alkali solubility is 50.53% lower than that of the damaged hair sample, and the alkali solubility is 11.26, which is close to that of the healthy hair sample.

Hydrolysed keratin selected in the experiment can not only penetrate the outer layer, but can also penetrate the stratum corneum and cortex for in-depth repair [6]. Malinauskyte, E. et al. have also made it clear in their research that low-molecular-weight keratin can be effectively repaired inside and outside the hair [20]. CeMeCo is a cell membrane complex, which is located in the gap between the hair cuticle and the cortical layer, and the content of amino acid residues is high. The addition of serine allows for the repair of more damaged areas not repaired by macromolecular keratin. The addition of serine not only repairs the cortical layer and the stratum corneum, but also may improve the CMC structure at the same time, prevent the stratum corneum from being damaged and falling off, make the hair fibre structure more stable, and reduce the alkali solubility. The damaged condition of silk is repaired, which makes the hair healthier and the anti-damage ability is improved.

### 3.4. Thermal Stability

**Figure 4:**
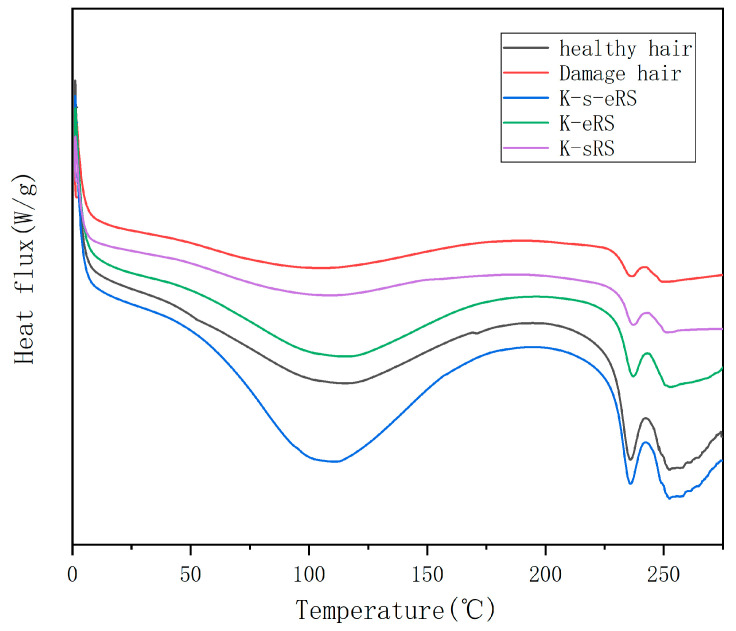
Thermal stability of perm and repair hair samples.

DSC is used to evaluate the thermal stability of the heating process of hair fibres, and it can effectively evaluate the thermal stability of the protein structure in hair fibres. Keratin-serine plus enzyme repair solution can improve the structure of hair silk protein and affect the thermal stability of hair silk protein. The peak between 230 and 240 °C is the endothermic peak of the hair sample, and the peak direction of the endothermic direction is downward, as shown in Figure 4.

The peak area of the damaged hair sample was the smallest, the α-keratin was seriously damaged, and the peak area of the keratin-serine plus enzyme-modified hair sample was the largest, indicating that keratin and amino acids infiltrated into the hair fibre cortex [21], α-keratin was repaired, thermal stability was stronger, and the degree of damage was reduced. Keratin-serine repair solution and keratin plus enzyme repair solution can also improve the thermal stability of hair samples to a certain extent, but the peak area is quite different from that of keratin-serine plus enzyme repair, which corresponds to the results of structural improvement and alkali solubility of hair samples. Improved thermal stability can better resist heat damage from sun exposure and hair dryers or curling bars. Even if the hair is in a high temperature, the moisture can be well locked and the loss of the moisture is reduced, so that the hair is not easy to dry and is more beautiful.

### 3.5. Mechanical Properties

The breaking stress refers to the maximum tensile force that can be endured by the cross-sectional area of the fibre. It is an index to measure the relative strength of the material and also one of the indexes to measure the tensile strength of the fibre. The fracture strain is the plastic strain of the deformation of the object under the action of the external force. Hair is a protein fibre, and the lack of fracture stress and fracture strain is one of the indicators of elastic healthy hair. After cold blanching treatment, hair becomes easy to break and deform, so the fracture stress and fracture strain decrease. In keratin-serine repair, keratin and serine are combined with damaged hair by simple physical attachment or weak bonds, which has little effect on the improvement of the hair’s physical properties after cleaning. The effect of keratin-serine plus enzyme repair on the physical properties of hair samples was more obvious than that of keratin plus enzyme repair, as shown in Table 2. On the one hand, serine is the conditioner in the process of hair care, which strengthens the CMC structure, and the cortex layer is closely combined with the stratum corneum. On the other hand, wool hydrolysed keratin provides exogenous α-keratin for the cortex layer, and the damaged and missing parts are repaired. The most important thing is that the TG enzyme cross-links keratin and serine to hair silk protein, forms amide bonds to improve the firmness of the protein network, repairs the cortex layer [22], improves the crystallinity of hair silk, and makes hair silk. The resistance and elasticity are significantly enhanced, and the damaged hair can be restored to the level of healthy hair samples through repair. The physical properties of the hair are improved, the resistance and elasticity of the hair are enhanced, the hair is stronger and stronger, and the vulnerability and the frangibility are reduced in daily combing.

### 3.6. Morphological Characterisation

**Figure 5:**
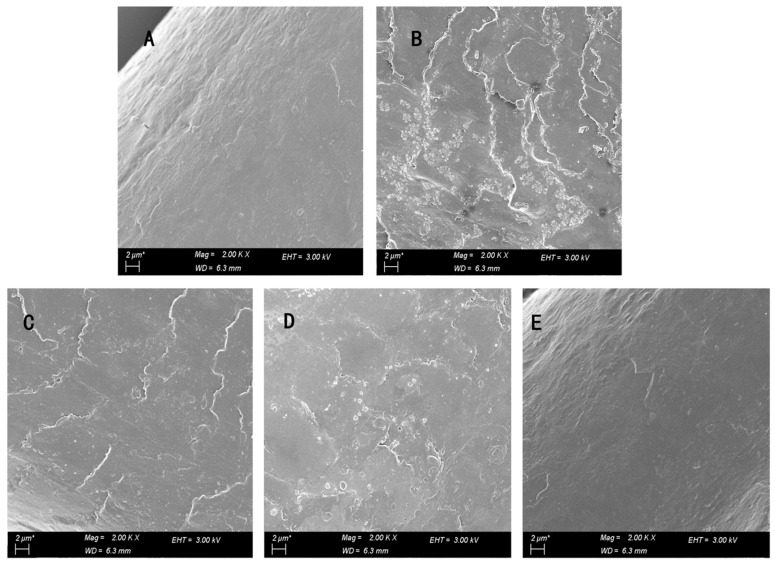
Electron scanning electron microscope photographs of different hair samples: (**A**): healthy hair; (**B**) Damaged hair; (**C**) K–s-RS; (**D**) K–eRS; (**E**) K–s-eRS.

Figure 5 shows scanning electron microscope photos of healthy hair samples, scalded hair samples, and hair samples treated with different repair fluids. It can be seen that the surface of the hair sample after scalding is rough, the outer structure is destroyed, the hair scales are warped, and the edges are irregular. After treatment with the keratin-serine repair solution and keratin plus enzyme repair solution, the damaged parts were repaired to a certain extent, and the hair scales were relatively flat, but there were still damaged hair scales that were not repaired. The hair sample repaired by keratin-serine plus enzyme was close to the morphology of the healthy hair sample, the hair scale was basically closed, the hair silk structure was complete, and the surface was smooth. The damaged defects are supplemented and combined with keratin, and meanwhile crosslinked and repaired by small molecules of serine, thus improving the overall hair quality. After the fur scales are closed, the invasion of harmful substances can be reduced, and the fur scales can be better repaired to better protect the cortical layer and reduce damage to the cortical layer from the outside, thereby enhancing the strength and toughness of the whole hair.

### 3.7. Damage Prevention

**Figure 6:**
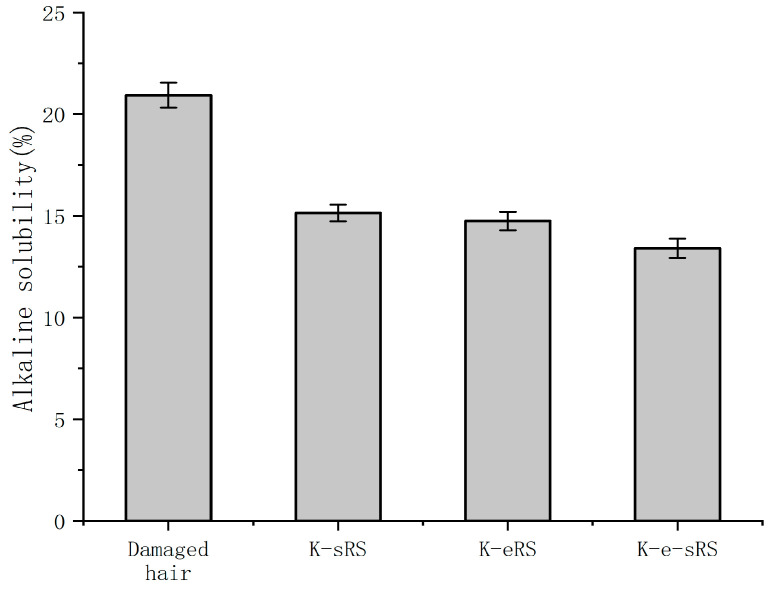
The alkaline solubility of damaged samples and damaged samples after curing.

Through Figure 6, it can be seen that after the curing of the three repair fluids, the hair fibre has a good ability to resist chemical damage. After being cured and then subjected to cold blanching treatment, the alkali solubility of the hair fibre is significantly reduced. The three repair fluids have a certain protective effect on the hair fibre, and the keratin-serine plus enzyme repair fluid has the best protective effect. The wool-hydrolysed keratin and serine in the keratin-serine plus enzyme curing solution form a dense protective film on the outside of the hair fibre, which can improve the ability of the cortical layer to resist external damage. At the same time, under the catalysis of the TG enzyme, it is closely combined with the hair, which enhances the stability of the cuticle structure, improves the resistance of the hair fibre, and effectively prevents and reduces the damage caused by the outside world. In particular, it can reduce chemical damage to the hair. The other two repair solutions can protect hair on the surface and inside of hair with adhesion and weak bonds to prevent chemical damage, but due to the lack of the TG enzyme, the protection method is not firm and the prevention ability is lower than that of keratin-serine plus enzyme repair solution.

### 3.8. Persistence

**Figure 7:**
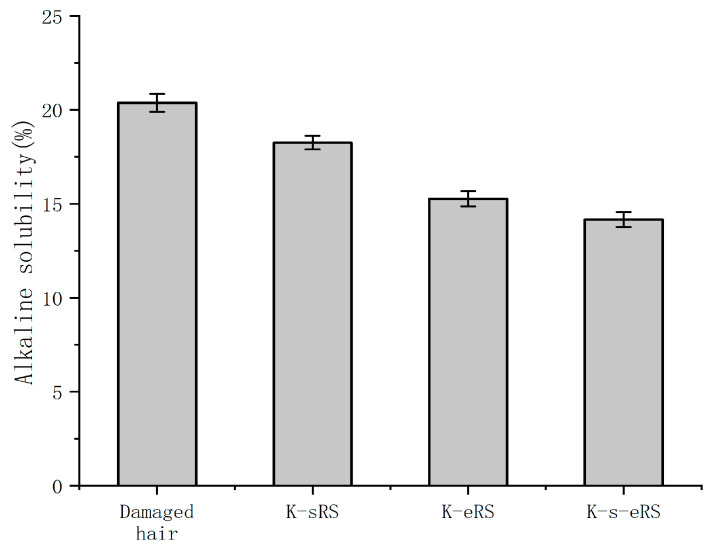
The alkaline solubility of damaged hair samples after repair after five washes.

The hair sample of the keratin-serine repair solution has a large alkali solubility after five times of cleaning, and cannot achieve the ‘quasi-permanent’ effect. Due to the lack of the TG enzyme, most of the effective components in the hair care solution cannot be firmly combined inside the hair fibres, and only a protective film can be formed outside the cortex layer, which can be easily eluted. The presence of the TG enzyme is very important for the ‘quasi-permanent’ effect. The addition of the TG enzyme can make the wool-hydrolysed keratin better bind to the damaged part of the hair fibre, and can achieve effective repair even after multiple cleanings. Compared with the keratin plus enzyme repair solution, the small molecule serine in the keratin-serine plus enzyme repair solution is combined with the part where it is difficult for the macromolecular keratin to enter, and the damaged part of the hair fibre is more comprehensively repaired, the washing resistance is enhanced, and the hair fibre can be effectively repaired for a long time. Even if the hair care times are reduced, the hair can maintain a good gloss and smoothness, as shown in Figure 7.

### 3.9. Moisture Retention

**Figure 8:**
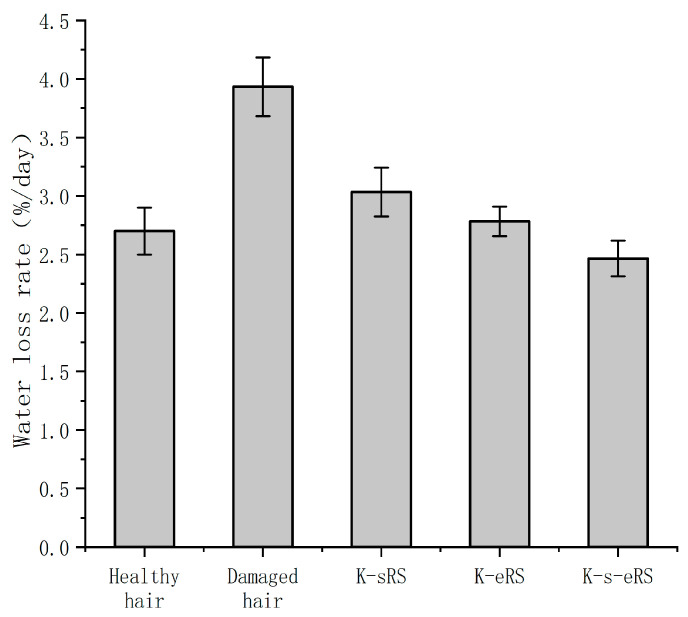
Water loss rate of different hair samples.

An increase in water content can lead to damaged hair silk becoming smoother, reducing the trouble of hair dryness after perming or washing, and hair care can improve the health of hair silk and reduce the rate of hair water loss. The water loss rate of the hair sample after different treatments is shown in Figure 8.

The structure of hair is damaged after cold blanching, and the water loss is serious. The loss of water in hair will cause dry hair. The keratin and serine in the keratin-serine repair solution can play an effective moisturizing effect, but it is easy to lose after cleaning; the presence of the TG enzyme in the keratin plus enzyme repair solution makes the wool-hydrolysed keratin well bound to the damaged site, but the keratin has a large molecular weight, and the locking water moisturizing effect is limited; in the keratin-serine plus enzyme repair solution, the enzymatic grafting or crosslinking of amino acids, keratin, and hair fibroin forms a network structure. When repairing the damaged part, it can be combined with water to lock water better. The membrane structure formed by the crosslinking component on the surface of the fibre can also prevent the loss of hair water, thereby increasing the internal water content. Barba, C. et al. [23] repaired the hair with wool keratin and wool peptide, and measured the increased breaking stress and breaking strain, which is related to the increased water content of the hair. The water retention property test in this experiment proved that the water-locking ability of hair fibre was enhanced, and the increase in water content was conducive to the improvement in the mechanical properties of hair fibre, which corresponded to the results of fracture stress and fracture strain. In this experiment, the water loss rate of hair repaired with the keratin-serine and enzyme repair liquid after 24 h was less than 3%. The water loss rate of the hair samples repaired by Zhang et al. [24] with 5% rabbit hair keratin was about 3.5% after 24 h, and the moisturizing effect of keratin-serine plus enzyme repair solution was better than that of the samples. The reduction in moisture loss can make the damaged hair more silky and smooth, alleviate the trouble of manic hair after perming, and effectively prevent the static electricity phenomenon caused by manic hair.

## 4. Conclusions

Compared with traditional hair care products that add only keratin or amino acids, the keratin-serine–enzyme repair solution described here offers the possibility of fundamentally improving and repairing the hair structure; in this method, keratin and serine form a protective film through the catalytic reaction of the TG enzyme, which can repair the main damaged parts of hair with inner keratin and supplement α keratin in the damaged cortex. Serine takes advantage of its small molecular weight to combine and repair the damaged parts in fine places, thus changing the physical and chemical properties of hair, increasing the content of amide bonds in damaged hair, improving the crystallinity of damaged hair, enhancing the thermal stability of damaged hair, and repairing the scales on the surface, making the appearance of damaged hair smoother, reducing the water loss rate of damaged hair, and making the hair more supple, shiny, healthy, and beautiful. Products of this type not only effectively repair damaged hair but also prevent damage to healthy hair samples. The biological repair liquid is ecologically friendly and safe, clean, and pollution-free, and the repair effect is remarkable and long-lasting.

## Figures and Tables

**Table 1:** Different crystallinity.

	Damaged Hair	K-sRS Hair Sample	K-eRS Hair Sample	K-s-eRS Hair Sample
Crystallinity (%)	21.91	15.01	16.57	13.76

**Table 2:** Different sample mechanical test results.

	Healthy Hair	Damaged Hair	K-sRS Hair Sample	K-eRS Hair Sample	K-s-eRS Hair Sample
Fracture stress (N)	2.014	1.031	1.337	1.648	1.806
Fracture strain (mm)	20.73	9.51	12.87	15.63	19.88

## Data Availability

All generated and analysed data used to support the findings of this study are included within the article.

## References

[1] Kajiura Y., Watanabe S., Itou T., Nakamura K., Iida A., Inoue K., Yagi N., Shinohara Y., Amemiya Y. (2006). Structural analysis of human hair single fibres by scanning microbeam SAXS. J. Struct. Biol..

[2] Velasco M.V.R., Dias T.C.D.S., Freitas A.Z.D., Júnior N.D.V., Pinto C.A.S.D.O., Kaneko T.M., Baby A.R. (2009). Hair fiber characteristics and methods to evaluate hair physical and mechanical properties. Braz. J. Pharm. Sci..

[3] Wolfram L.J. (2003). Human hair: A unique physicochemical composite. J. Am. Acad. Dermatol..

[4] Song K., Xu H., Xie K., Yang Y. (2016). Effects of chemical structures of polycarboxylic acids on molecular and performance manipulation of hair keratin. RSC Adv..

[5] Tinoco A., Martins M., Cavaco-Paulo A., Ribeiro A. (2022). Biotechnology of functional proteins and peptides for hair cosmetic formulations. Trends Biotechnol..

[6] Cruz C.F., Azoia N.G., Matamá T., Cavaco-Paulo A. (2017). Peptide—Protein interactions within human hair keratins. Int. J. Biol. Macromol..

[7] Watt I.C., Morris R. (1969). Factors affecting the ther-mal contraction of keratin fibres. J. Polym. Sci. Pol..

[8] Barba C., Martí M., Roddick-Lanzilotta A., Manich A., Carilla J., Parra J., Coderch L. (2010). Effect of wool keratin proteins and peptides on hair water sorption kinetics. J. Therm. Anal. Calorim..

[9] Villa A.L.V., Aragão M.R.S., Dos Santos E.P., Mazotto A.M., Zingali R.B., De Souza E.P., Vermelho A.B. (2013). Feather keratin hydrolysates obtained from microbial keratinases: Effect on hair fiber. BMC Biotechnol..

[10] Gaspar A.L.C., de Góes-Favoni S.P. (2015). Action of microbial transglutaminase (MTGase) in the modification of food proteins: A review. Food Chem..

[11] Soun B., Kaur D., Jose S. (2020). Effect of Transglutaminase Enzyme on Physico-mechanical Properties of Rambouillet Wool Fiber. J. Nat. Fibers.

[12] Cardamone J.M., Phillips J.G. (2007). Enzyme-mediated Crosslinking of Wool. Part II: Keratin and Transglutaminase. Text. Res. J..

[13] Lin Z. (2016). Study on mTG Enzyme-Catalyzed Reinforcement of Fragile Wool Fabrics. Ph.D. Thesis.

[14] Li Z., Xiao J. (2021). Repair of damaged hair protein fiber by jointly using transglutaminase and keratin. Scienceasia.

[15] Gillespie J.M., Haylett T., Lindley H. (1968). Evidence of homology in a high-sulphur protein fraction (SCMK-B2) of wool and hair alpha-keratins. Biochem. J..

[16] Segal L. (1959). An Empirical Method for Estimating the Degree of Crystallinity of Native Cellulose Using the X-ray Diffractometer. Text. Res. J..

[17] The Textile Journal Group (2006). Total Colour Management in Textiles.

[18] Wojciechowska E., Włochowicz A., Wesełucha-Birczyńska A. (1999). Application of fourier-transform infrared and raman spectroscopy to study degradation of the wool fiber keratin. J. Mol. Struct..

[19] Zhang P., Zhang N., Wang Q., Wang P., Yuan J., Shen J., Fan X. (2018). Disulfide bond reconstruction: A novel approach for grafting of thiolated chitosan onto wool. Carbohydr. Polym..

[20] Malinauskyte E., Shrestha R., Cornwell P.A., Gourion-Arsiquaud S., Hindley M. (2021). Penetration of different molecular weight hydrolyzed keratins into hair fibers and their effects on the physical properties of textured hair. Int. J. Cosmet. Sci..

[21] Fernandes M., Cavaco-Paulo A. (2012). Protein disulphide isomerase-mediated grafting of cysteine-containing peptides onto over-bleached hair. Biocatal. Biotransform..

[22] Ribeiro A., Matamá T., Cruz C.F., Gomes A.C., Cavaco-Paulo A.M. (2013). Potential of human γD-crystallin for hair damage repair: Insights into the mechanical properties and biocompatibility. Int. J. Cosmet. Sci..

[23] Barba C., Scott S., Roddick-Lanzilotta A., Kelly R., Manich A.M., Parra J.L., Coderch L. (2010). Restoring Important Hair Properties with Wool Keratin Proteins and Peptides. Fibers Polym..

[24] Yi Z.R., Hao Z., Li S. (2018). Preparation of rabbit hair keratin and its application in sunscreen cosmetics. Res. Dev. Nat. Prod..

